# Trends in Stroke Prevention between 2014 and 2018 in Hospitalized Atrial Fibrillation Patients

**DOI:** 10.1155/2021/6657776

**Published:** 2021-02-08

**Authors:** B. Bielecka, I. Gorczyca, O. Jelonek, B. Wożakowska-Kapłon

**Affiliations:** ^1^1st Clinic of Cardiology and Electrotherapy, Swietokrzyskie Cardiology Center, Kielce 25-736, Poland; ^2^Collegium Medicum, The Jan Kochanowski University, Kielce 25-369, Poland

## Abstract

In recent years, significant changes in stroke prophylaxis in patients with atrial fibrillation (AF) have been observed. Non-vitamin K antagonist oral anticoagulants (NOACs) are more commonly used in the prevention of thromboembolic complications in patients with AF. The aim of the study was to evaluate recommended stroke prophylaxis in patients with AF and to identify predictors of using NOACs in patients treated with anticoagulant therapy. The present study was a retrospective, observational, single-center study which included consecutively hospitalized patients in the reference cardiology center from January 2014 to December 2018. In the study group of 4027 patients with AF, to prevent thromboembolic complications, OACs were used in 3680 patients (91.4%), an antiplatelet drug(s) was used in 124 patients (3.1%), and 223 patients (5.5%) did not undergo any thromboembolic event prevention. In the group of 3680 patients treated with OACs, 2311 patients (62.8%) received NOACs and 1639 patients (37.2%), VKAs. Independent predictors of the use of NOACs were age (OR, 1.02; 95% CI, 1.01–1.03; *P* < 0.001), a previous thromboembolic event (OR, 1.29; 95% CI, 1.01–1.65; *P*=0.04), nonpermanent AF (OR, 1.61; 95% CI, 1.34–1.93; *P* < 0.001), and eGFR (OR, 1.22; 95% CI, 1.02–1.46; *P*=0.03). Between 2014 and 2018, an increase of patients treated with OACs, mainly with NOACs, was observed. Age, past thromboembolic complications, nonpermanent AF, and preserved renal function determined the choice of NOACs.

## 1. Introduction

Atrial fibrillation (AF) is the most frequent supraventricular arrhythmia. It affects roughly 1-2% of the population and is connected with a fivefold increase of a thromboembolic complication risk [1, 2]. It is estimated that among middle-aged people every fourth person will suffer from AF. The risk of thromboembolic complications in patients with AF is not homogeneous and depends on age, sex, and comorbidities. To estimate the risk of thromboembolic complications in patients with AF, the CHA_2_DS_2_-VASc score is used [3]. The guidelines of the European Society of Cardiology point that anticoagulant therapy is advised in patients with a high risk of thromboembolic complications. Anticoagulant prophylaxis should not be applied in patients of low risk of thromboembolic complications [4]. Vitamin K antagonists (VKAs) and non-vitamin K antagonist oral anticoagulants (NOACs) appeared to be effective in the prevention of thromboembolic complications in patients with AF [5–8]. In recent years, it has been possible to observe a change in stroke prophylaxis in AF patients, which results from more and more common NOAC administration in the prevention of thromboembolic complications [9, 10]. Although the guidelines clearly define indications for OAC application, it is not easy to implement them in clinical practice. In international registries, both patients of low thromboembolic risk treated with OACs and patients of high thromboembolic risk not receiving OACs are observed [11–13].

The aim of the study was to evaluate recommended anticoagulant prophylaxis in patients with AF, with a particular reference to the assessment of NOAC application frequency and an attempt to identify factors that clinched the use of NOACs in patients treated with OACs.

## 2. Materials and Methods

### 2.1. Study Group

The study includes all consecutive patients with AF hospitalized in a referential cardiology center from January 2014 to December 2018, who were hospitalized for urgent and planned reasons. Patients were included if they were at least 18 years of age and had a history of AF documented by electrocardiography or in their medical history.

Mechanical heart valve, moderate or severe mitral valve, and death during hospitalization were the exclusion criteria.

AF was diagnosed on the basis of the definition of the European Society of Cardiology, according to which arrhythmia can be identified using an electrocardiogram showing irregular atrial rhythm lasting longer than 30 seconds [3].

### 2.2. Assessed Parameters

Baseline characteristics concerning medical history, AF type, demographics, diagnostic test results, and pharmacotherapy were collected.

CHA2DS2-VASc (congestive heart failure, hypertension, age ≥75 years, diabetes mellitus, stroke/transient ischaemic attack, vascular disease, age 65–74 years, and sex category) score was used to make an assessment of thromboembolic risk whereas bleeding risk was defined with HAS-BLED (hypertension, abnormal renal/liver function, stroke, bleeding, labile INR, elderly (>65 years), and drug/alcohol consumption) [13].

The estimated glomerular filtration rate (eGFR) used to assess patients' kidney function was calculated using the Modification of Diet in Renal Disease (MDRD) Study equation.

The study was approved by the Ethics Committee of the Swietokrzyskie Medical Chamber in Kielce. Informed consent from the patients was not required by the Ethics Committee.

### 2.3. Management of Antithrombotic Therapy

Antithrombotic therapy recommended during the patients' discharge from the hospital was evaluated. Three types of regiments were defined: OAC ± antiplatelet drug (AP), AP alone, and no antithrombotic treatments. OAC group included VKAs, apixaban, dabigatran, and rivaroxaban alone or with AP. Edoxaban has been registered in Europe as a drug for preventing thromboembolic complications in patients with AF; however, it is not available in Poland. AP group included acetylsalicylic acid (ASA) or/and clopidogrel, ticagrelor, and prasugrel. No antithrombotic treatment was defined by the absence of OAC and AP prescription.

### 2.4. Statistical Analysis

The normality of data distributions was tested using the Kolmogorov–Smirnov test. The hypothesis of normality was rejected for most of the analysed variables; therefore, nonparametric methods were used. The level of significance was set as *α* = 0.05. Descriptive statistics are presented as means and standard deviation or median and interquartile range. The distribution of qualitative data was presented as frequency and percentages. Frequencies were compared using the *χ*^2^ test. The distributions of quantitative variables were compared using the Mann–Whitney *U* test. The odds ratios (OR) together with a 95% confidence interval (CIs) were determined using a univariate logistic regression model. A multivariate logistic regression analysis was used to explore the variables associated with increasing the chances of using non-vitamin K antagonist oral anticoagulants (NOACs); the variables which presented statistically significant OR were included. Statistical analyses were performed with SPSS version 25 (IBM).

## 3. Results and Discussion

### 3.1. Characteristics of the Study Group

In the study group of 4027 patients with AF, the mean age was 71.7 (11.4) years; 42.6% were women. Most often comorbidities were hypertension—3117 patients (77.4%), heart failure—2520 patients (62.6%), and vascular disease—1482 patients (36.8%). Nonpermanent AF was present in 2549 patients (63.3%).

A high risk of thromboembolic complications according to the CHA_2_DS_2_-VASc was reported in 3630 patients (90.1%), and a high risk of bleeding complications according to the HAS-BLED was reported in 1179 patients (29.3%).  Table 1 presents the clinical characteristics in the study cohort.

### 3.2. Prevention of Thromboembolic Complications

In the study group of 4027 patients with AF, OACs were used in 3680 patients (91.4%), antiplatelet drug/drugs in 124 patients (3.1%), and 223 patients (5.5%) did not undergo any thromboembolic event prevention.

In the group of patients treated with OACs, 1369 of them (37.2%) were administered VKAs and 2311 (62.8%) NOACs. In the group receiving NOACs, dabigatran was used in 51.4% of patients, rivaroxaban in 34.6%, and apixaban in 14% of them.

**Figure 1:**
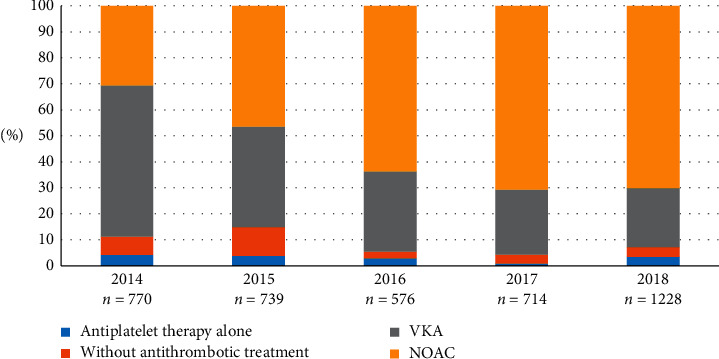
Stroke prophylaxis in patients with atrial fibrillation in particular years of hospitalization.

**Figure 2:**
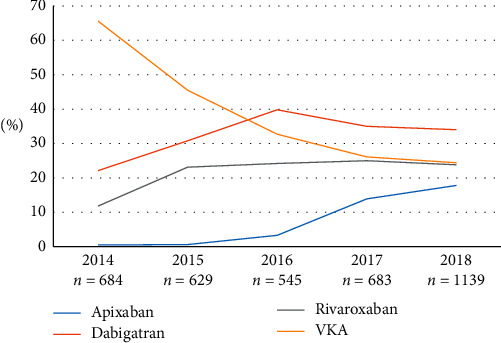
Proportion of patients treated with oral anticoagulants in particular years of hospitalization.

Between 2014 and 2018, the increase in the proportion of NOAC-treated patients was observed from 30.5% to 70.1% (Figure 1).  Figure 2 presents the proportion of patients treated with OACs in particular years of hospitalization.

**Figure 3:**
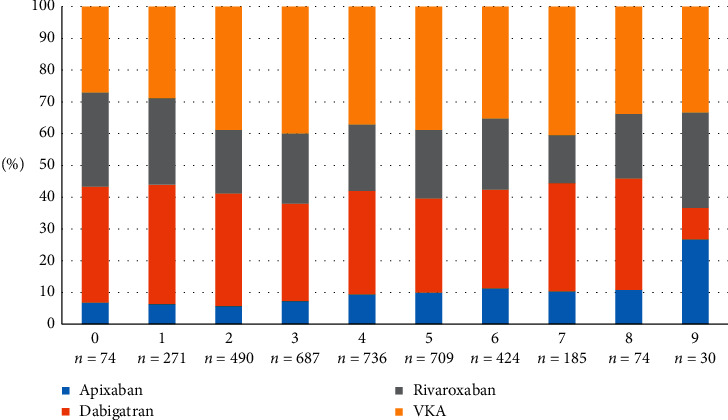
Prescription pattern of oral anticoagulants based on CHA2DS2-VASc score.

Figure 3 shows the proportion of patients treated with individual OACs in particular categories according to the CHA_2_DS_2_-VASc score.

### 3.3. Comparison of Patients Treated with VKAs and NOACs

In the group of 3680 patients treated with OACs, 2311 patients (62.8%) received NOACs and 1639 of them (37.2%) were administered VKAs. NOAC patients in comparison to VKA patients were older [72.2(11.5) vs. 71.3(10); *P*=0.001] and more often diagnosed with past thromboembolic complications (13.9% vs. 11.6%; *P*=0.047) and nonpermanent AF (68.8% vs. 52%; *P* < 0.001). The CHA_2_DS_2_-VASc score was similar in patients treated with VKAs and NOACs [3.9(1.9) vs. 4(1.8); *P*=0.34] whereas the HAS-BLED score was higher in VKA patients compared with NOAC ones [2(1) vs. 2(0.9); *P*=0.005]. The eGFR<60 ml/min/1.73m2 statistically significantly more often occurred in the group of VKA patients than in the NOAC one (67.1% vs. 62.4%; *P*=0.008). Patients treated with VKAs more often than NOAC patients were administered antiplatelet pharmaceutical/pharmaceuticals (9.7% vs. 6.1%; *P* < 0.001) (Table 2).

### 3.4. Predictors of NOAC Choice in the Group of Patients Receiving OACs

In the univariable logistic regression analysis, numerous predictors of NOAC prescription were found (Table S1). In the multivariable model, factors associated with the selection of NOACs versus VKAs included the following: age, eGFR, a previous thromboembolic event, nonpermanent AF, left atrial (LA) diameter and antiplatelet drug/drugs.

Table 3 shows the predictors of the NOAC use—age (OR, 1.02; 95% CI, 1.01–1.03; *P* < 0.001), a previous thromboembolic event (OR, 1.29; 95% CI, 1.01–1.65; *P*=0.04), nonpermanent AF (OR, 1.61; 95% CI, 1.34–1.93; *P* < 0.001), and eGFR (OR, 1.22; 95% CI, 1.02–1.46; *P*=0.03).

LA diameter (OR, 0.97; CI, 0.96–0.98; *P* < 0.001) and antiplatelet drug/drugs (OR, 0.68; CI, 0.5–10.92; *P*=0.01) diminished the chance of choosing NOACs.

## 4. Discussion

Many studies have reported general improvement of AF patient management in terms of OAC treatment after NOACs were introduced [14, 15]. The same applies to the steady decrease in VKA and antiplatelet drug use in favour of NOAC therapies. Similar results were achieved in the present study, which in particular demonstrates new data concerning prescription of OACs as well as NOACs and VKAs in AF patients from Poland.

In recent years, using OACs in AF treatment gradually increased worldwide (>80%). However, regional differences are clearly visible—the highest uptake in Europe (90%) and North America (78.2%) and the lowest one in Asia (57.4%). Interestingly, the percentage of patients receiving only antiplatelet drugs or untreated decreased, even if it was still high in the patients of high risk [16]. In the presented group of 4027 patients with AF, 91% of them received OACs. In the All Nippon AF in the Elderly (ANAFIE) Registry, 92% of 32726 patients aged ≥ 75 years were administered OACs [17]. Similarly, the Atrial Fibrillation in Octogenarians (OCTOFA) Study showed that 92% of 738 patients ≥ 80 years received OACs [18]. In the EURObservational Research Programme on Atrial Fibrillation (EORP-AF) Long-Term General Registry, OACs were used in 84.9% of patients [19].

In the present study, NOACs were used in 63% of patients treated with OACs, and a significant increase in the proportion of patients treated with NOACs was observed over five years. In 2014, they constituted 34.4% of OACs and in 2018 it was 75.6%. Many studies clearly outline a significant implementation of NOACs in thromboembolic prophylaxis of AF in recent years. In Global Anticoagulant Registry in the FIELD-Atrial Fibrillation (GARFIELD-AF), the proportion of prescribed NOACs rose from 34% to 62% within 3 years [20]. EORP-AF General Long-Term General Registry in comparison to EORP-AF Pilot showed that during four years there was a NOAC prescription increase from less than 10% of patients to about 35% [19, 21]. From 2011 to 2016, Balsam et al. observed an increase of prescribed NOACs to 2/3 of all OACs [22].

In the present study, dabigatran was the most frequently prescribed NOAC. Dabigatran was the first, rivaroxaban the second, and apixaban the third NOAC available in Poland, and all the drugs were available in the whole study period. Our study showed a strong increase in apixaban use since its introduction. It was higher than the increase in dabigatran and rivaroxaban use.

In the present study, antiplatelet drug was received by 3.1% of patients (124 out of 4,027), and 5.5% of patients were discharged from a hospital without any kind of anticoagulant therapy. Within 5 years, the proportion of patients with AF treated with antiplatelet pharmaceuticals decreased from 4.2% to 3.4%, and patients without anticoagulant prophylaxis decreased from 7% to 3.8%. According to the BLITZ-AF study, 9.1% of patients received only antiplatelet drugs and 7.5% were not treated with any antithrombotic drugs [23]. There are similar results from the Global Registry on Long-Term Oral Antithrombotic Treatment in Patients with Atrial Fibrillation Registry (GLORIA-AF) where 12.1% were on antiplatelets and 7.8% were not on antithrombotic therapy [24]. In light of the effectual guidelines related to AF, antiplatelet pharmaceuticals should not be used in the prevention of thromboembolic complications in patients with AF [4]. However, it happens that they are used against the guidelines in situations where OAC administration seems to be too risky.

In our study, we identified independent predictors of NOAC use and it is a novel finding of specific interest. In the present study, the predisposing factors for the use of NOACs among AF patients were age, eGFR, a previous thromboembolic event, nonpermanent AF, while the predisposing factors for VKA use were LA diameter and antiplatelet drug/drugs. Although the current guidelines make no distinction between nonpermanent and permanent AF for stroke prevention, clinical data confirmed that the type of AF was associated with an increased risk of stroke [25]. It is possible then that, in the future, the AF type will be included in a thromboembolic risk stratification and choosing anticoagulant prophylaxis. In the present study, the strongest predictor of NOAC vs. VKA choice was nonpermanent AF. Similarly, in the group of patients in GARFIELD-AF Registry, NOACs were preferred in patients with paroxysmal AF [26]. Choosing NOACs in patients with nonpermanent AF is connected with the fact that they are usually de novo AF patients and when there is a decision to use OACs, according to the guidelines, NOACs should be preferred [4].

NOACs are preferred after a stroke and other thromboembolic events [4]. In the present study, a previous thromboembolic event was a strong predictor for the choice of NOAC. Thromboembolic events were observed in 13% of patients with AF. A similar proportion of patients with thromboembolic complications was noticed in PREvention of thromboembolic events–European Registry in Atrial Fibrillation (PREFER) Registry—8.4% of patients [27]. Gorczyca et al. [28] showed that in AF patients after a thromboembolic event, 59% of patients on OACs were treated with NOACs. The Novel Oral Anticoagulants in Stroke Patients (NOACISP LONG-TERM) Registry showed that NOACs were used in 78% of patients treated with OACs in the secondary prevention of thromboembolic events [29].

Impaired renal function is an acknowledged risk factor of thrombus formation, stroke, systemic embolism, and bleeding events. Data showed that NOACs would reduce the risk of stroke or systemic embolism and major bleeding concerning different levels of renal function [30–32]. In the present study, it was shown that higher eGFR predisposes the choice of NOACs. NOACs are contraindicated in patients with significant impairment of renal function.

Elderly patients more often choose NOACs in the study group which complies with the GARFIELD Registry results [26] and the ANAFIE study [17]. In older patients with AF, NOACs have a better efficacy and safety profile than VKAs [33]. In contrast, the results from the Outcomes Registry for Better Informed Treatment of Atrial Fibrillation II (ORBIT-AF II) showed that patients treated with NOACs were younger than patients treated with VKAs [34]. It is difficult to compare the data obtained from different registries because their results are dependent on the clinical characteristics of patients and the preferences of doctors. The data depends also on the geographical region.

In the present study, LA extension was a predictor of VKA usage. It is possible that patients with extended LA had longer-lasting AF than patients with the normal LA diameter and in these patients, VKAs have been administered for many years.

A factor predisposing the choice of VKA in the prevention of thromboembolic complications was also the application of antiplatelet drug/drugs. Balsam et al. [22] showed that VKA patients were more often treated with antiplatelet pharmaceuticals than patients receiving NOACs. Similarly, in the GARFIELD Registry, use of antiplatelet medications was a predictor of choosing VKAs; however, interestingly, acute coronary syndrome clinched the choice of NOAC [26]. It seems that, in clinical practice, adjusting the NOAC dose to a clinical risk and benefit balance may cause some problems when using NOACs and antiplatelet drugs concomitantly. The current guidelines regarding chronic coronary syndromes allow prescribing the above in a full or reduced dose when using dabigatran and rivaroxaban, depending on the evaluation of individual haemorrhagic and ischemic risks [35]. In the present study, cancer was not a predictor of anticoagulant choice. Mariani et al. [36] showed that NOACs, in comparison to VKA, were associated with a significant reduction of the rates of thromboembolic events and major bleeding complications in AF patients with cancer.

## 5. Limitations

This study has several limitations. Firstly, it was not possible to assess anticoagulation effects on outcomes due to collected data observational nature and no follow-up data. Secondly, the present study is a single-center registry, but it was conducted in the referential center, where ambulatory patients were sent, and in other hospitals. Thirdly, in the present study, most patients with AF were not OAC-naïve. Thus, despite the unicentric character of the study, it shows the anticoagulant therapy trends of doctors from particular regions referring patients to the center where the study comes from.

## 6. Conclusions

In the present “real-world” study, significant changes in the prophylaxis of thromboembolic complications in patients with AF within recent years were reported. The number of patients treated with OACs increased significantly. In most of them, NOACs were administered. Age, previous thromboembolic complications, nonpermanent AF, and preserved renal function determined the choice of NOACs in patients treated with OACs.

## Figures and Tables

**Table 1:** Baseline characteristics of the study group.

Variable	All *n* = 4027	OAC *n* = 3680	APT *n* = 124	None *n* = 223
Female, *n* (%)	1714 (42.6)	1572 (42.7)	45 (36.3)	97 (43.5)
Age, mean (SD), years	71.7 (11.4)	71.8 (12)	71.4 (13.4)	70.2 (15.5)

*Clinical characteristics, n (%)*				
Heart failure	2520 (62.6)	2276 (61.8)	85 (68.5)	159 (71.3)
Hypertension	3117 (77.4)	2891 (78.6)	79 (63.7)	147 (65.9)
Vascular disease	1482 (36.8)	1331 (36.2)	79 (63.7)	72 (32.3)
Diabetes mellitus	1148 (28.5)	1063 (28.9)	30 (24.2)	55 (24.7)
Previous thromboembolic event	514 (12.8)	480 (13.0)	14 (11.3)	20 (9)

*AF type, n (%)*				
Paroxysmal	1788 (44.4)	1566 (42.6)	99 (79.8)	123 (55.2)
Persistent	761 (18.9)	737 (20.0)	5 (4.1)	19 (8.5)
Permanent	1478 (36.7)	1377 (37.4)	20 (16.1)	81 (36.3)
Nonpermanent	2549 (63.3)	2303 (62.6)	104 (83.9)	142 (63.7)

*Thromboembolic risk*				
CHA2DS2-VASc mean (SD)	3.9 (1.8)	3.9 (1.8)	4.0 (1.8)	3.7 (2.0)
CHA2DS2-VASc = 0, *n* (%)	99 (2.5)	74 (2.0)	4 (3.2)	21 (9.4)
CHA2DS2-VASc = 1, *n* (%)	298 (7.4)	271 (7.4)	7 (5.6)	20 (9.0)
CHA2DS2-VASc ≥ 2, *n* (%)	3630 (90.1)	3335 (90.6)	113 (91.2)	182 (81.6)

*Bleeding risk*				
HAS-BLED, mean (SD)	2.0 (1.0)	2.0 (0.9)	2.0 (1.1)	2.0 (1.1)
HAS-BLED ≥ 3, *n* (%)	1179 (29.3)	1064 (28.9)	36 (29)	79 (35.4)

*Laboratory tests*				
HGB, g/dl	*n* = 3957	*n* = 3615	*n* = 124	*n* = 218
Mean (SD)	13.3 (1.8)	13.4 (1.7)	12.8 (2.3)	12.8 (2.5)
PLT, 103/ul	*n* = 3930	*n* = 3594	*n* = 121	*n* = 215
Mean (SD)	212.0 (75)	210.6 (72.8)	235.1 (88.2)	223.2 (95.7)
Creatinine, mg/dl	*n* = 4008	*n* = 3668	*n* = 123	*n* = 217
Mean (SD)	1.3 (0.5)	1.3 (0.4)	1.2 (0.4)	1.4 (0.8)
eGFR, 60 ml/min/1.73 m^2^	*n* = 4008	*n* = 3679	*n* = 123	*n* = 217
Mean (SD)	54.9 (16.5)	54.8 (16.3)	57.9 (16.4)	53.6 (20)
eGFR < 60 ml/min/1.73 m^2^, *n* (%)	2558 (63.8)	2355 (64.0)	71 (57.7)	68 (31.3)

*Echocardiography*				
LA, mm	*n* = 2944	*n* = 2667	*n* = 106	*n* = 171
Mean (SD)	46.9 (7.8)	47.1 (7.7)	43.8 (7.3)	44.7 (8.9)
LVDD, mm	*n* = 2960	*n* = 2680	*n* = 107	*n* = 173
Mean (SD)	52.9 (8.8)	53.1 (8.7)	51.8 (9.1)	50.7 (9.8)
LVEF, (%)	*n* = 2985	*n* = 2704	*n* = 108	*n* = 173
Mean (SD)	48.3 (16.7)	48.2 (17.1)	47.3 (11.6)	49.7 (14.3)

Data are presented as number (percentage) or mean (standard deviation) (SD) or median (interquartile range) (IQR). AF: atrial fibrillation; APT: antiplatelet drug/drugs; eGFR: estimated glomerular filtration rate; LA: left atrium; LVDD: left ventricular end-diastolic dimension; LVEF: left ventricular ejection fraction; and OAC: oral anticoagulant.

**Table 2:** Clinical characteristics of patients with atrial fibrillation treated with vitamin K antagonist and non-vitamin K antagonist oral anticoagulant.

Clinical feature	OAC *n* = 3680	NOAC *n* = 2311	VKA *n* = 1369	*P*
*Female, n (%)*	1572 (42.7)	1008 (43.6)	564 (41.2)	0.152
*Age, years*				0.001
Mean (SD)	71.8 (12)	72.2 (11.5)	71.3 (10)	
Median (IQR)	72 (15)	73 (16)	71 (14)	

*Clinical characteristics, n (%)*				
Heart failure	2276 (61.8)	1338 (57.9)	938 (68.5)	<0.001
Hypertension	2891 (78.6)	1804 (78.1)	1087 (79.4)	0.339
Diabetes mellitus	1063 (28.9)	618 (26.7)	445 (32.5)	<0.001
Previous thromboembolic event	480 (13)	321 (13.9)	159 (11.6)	0.047
Vascular disease	1331 (36.2)	800 (34.6)	531 (38.8)	0.011
Cancer	157 (4.3)	102 (4.4)	55 (4.0)	0.345

*AF type, n (%)*				
Paroxysmal	1566 (42.6)	1067 (46.2)	499 (36.4)	<0.001
Persistent	737 (20.0)	524 (22.7)	213 (15.6)	<0.001
Permanent	1377 (37.4)	720 (31.2)	657 (48.0)	<0.001
Nonpermanent	2303 (62.6)	1591 (68.8)	712 (52.0)	<0.001

*Thromboembolic risk*				
*CHA2DS2-VASC score*				0.338
Mean (SD)	3.9 (1.8)	3.9 (1.9)	4.0 (1.8)	
Median (IQR)	4.0 (2.0)	4.0 (2.0)	4.0 (2.0)	
CHA_2_DS_2_-VASC = 0, *n* (%)	74 (2)	54 (2.3)	20 (1.5)	0.002
CHA_2_DS_2_-VASC = 1, *n* (%)	271 (7.4)	193 (8.4)	78 (5.7)	
CHA2DS2-VASC ≥ 2, *n* (%)	3335 (90.6)	2064 (89.3)	1271 (92.8)	

*Bleeding risk*				
*HASBLED score*				0.005
Mean (SD)	2.0 (0.9)	2.0 (0.9)	2.0 (1.0)	
Median (IQR)	2.0 (2.0)	2.0 (2.0)	2.0 (2.0)	
HASBLED ≥ 3, *n* (%)	1064 (28.9)	621 (26.8)	443 (32.4)	0.048

*Laboratory tests*				
*HGB, g/dl*	*n* = 3615	*n* = 2268	*n* = 1347	0.091
Mean (SD)	13.4 (1.7)	13.4 (1.7)	13.3 (1.7)	
Median (IQR)	13.5 (2.2)	13.5 (2.3)	13.4 (2.1)	
*PLT, 103/ul*	*n* = 3594	*n* = 2255	*n* = 1339	<0.001
Mean (SD)	210.6 (72.8)	215.3 (75.5)	202.6 (67.1)	
Median (IQR)	200.0 (78.0)	205.0 (78.0)	193.0 (74.0)	
*Creatinine, mg/dl*	*n* = 3668	*n* = 2304	*n* = 1364	<0.001
Mean (SD)	1.3 (0.4)	1.2 (0.3)	1.3 (0.5)	
Median (IQR)	1.2 (0.4)	1.2 (0.3)	1.2 (0.4)	
*eGFR, 60 ml/min/1.73 m* ^*2*^	*n* = 3679	*n* = 2311	*n* = 1368	<0.001
Mean (SD)	54.8 (16.3)	55.8 (16.1)	53.3 (16.4)	
Median (IQR)	54.2 (21.1)	55.1 (21.3)	52.7 (20.7)	
eGFR < 60 ml/min/1.73 m^2^, *n* (%)	2355 (64.0)	1442 (62.4)	913 (67.1)	0.008

*Echocardiography*				
*LA, mm*	*n* = 2667	*n* = 1664	*n* = 1003	<0.001
Mean (SD)	47.1 (7.7)	46.1 (7.3)	48.8 (8.1)	
Median (IQR)	46.0 (10.0)	45.0 (9.0)	48.0 (10.0)	
*LVDD, mm*	*n* = 2680	*n* = 1676	*n* = 1004	<0.001
Mean (SD)	53.1 (8.7)	52.3 (8.4)	54.4 (9.0)	
Median (IQR)	52.0 (11.0)	51.0 (10.0)	53.0 (11.0)	
*LVEF, n (%)*	*n* = 2704	*n* = 1688	*n* = 1016	<0.001
Mean (SD)	48.2 (17.1)	49.5 (18.8)	46.1 (13.7)	
Median (IQR)	50.0 (20.0)	52.0 (18.0)	50.0 (19.0)	
*Antiplatelet drug/drugs, n (%)*	275 (7.5)	142 (6.1)	133 (9.7)	<0.001

Data are presented as number (percentage) or mean (standard deviation) (SD) or median (interquartile range) (IQR). AF: atrial fibrillation; eGFR: estimated glomerular filtration rate; HGB: hemoglobin concentration; LA: left atrium; LVDD: left ventricular end-diastolic dysfunction; LVEF: left ventricular ejection fraction; NOAC: non-vitamin K antagonist oral anticoagulant; OAC: oral anticoagulation therapy; PLT: platelet count; TIA: transient ischemic attack; and VKA: vitamin K antagonist oral anticoagulants.

**Table 3:** Factors increasing the chances of using non-vitamin K antagonist oral anticoagulant in analysed patients—multivariate logistic regression analysis.

Factors	OR	95% CI	*P*
Age (per year)	1.02	1.01–1.03	<0.001
Heart failure	0.84	0.70–1.00	0.057
Diabetes mellitus	0.88	0.74–1.05	0.163
Previous thromboembolism event	1.29	1.01–1.65	0.038
Vascular disease	0.91	0.77–1.09	0.320
Nonpermanent AF	1.61	1.34–1.93	<0.001
eGFR (per unit)	1.22	1.02–1.46	0.030
LA diameter	0.97	0.96–0.98	<0.001
Antiplatelet drug/drugs	0.68	0.51–0.92	0.011

AF: atrial fibrillation; CI: confidence interval; eGFR: estimated glomerular filtration rate; LA: left atrium; and OR: odds ratio.

## Data Availability

The source data used to support the findings of this study are available from the corresponding author upon request.

## Supplementary Materials

Supplementary Materials Table S1: factors increasing the chances of using non-vitamin K antagonist oral anticoagulants in analysed patients—univariable logistic regression analysis.
