# Supplementary material for: Trends in Stroke Prevention between 2014 and 2018 in Hospitalized Atrial Fibrillation Patients
Source: Cardiol Res Pract. 2021 Feb 8;2021:6657776. doi: 10.1155/2021/6657776 (PMC7886594; doi:10.1155/2021/6657776)
Supplement: Supplementary Materials — Table S1: factors increasing the chances of using non-vitamin K antagonist oral anticoagulants in analysed patients—univariable logistic regression analysis. [file 6657776.f1.docx]

**Table S1.** Factors increasing the chances of using non-vitamin K antagonist oral anticoagulants in analysed patients – univariable logistic regression analysis.

| **Factors** | **NOAC**  **n = 2311** | **VKA**  **n = 1369** | OR | 95% CI | p |
| --- | --- | --- | --- | --- | --- |
| **Age, years** | 72.2 (11.5) | 71.3 (10) | 1.00 | 1.00-1.01 | **0.019** |
| **Heart failure, n (%)** | | | | | |
| No | 973 (42.1) | 431(31.5) | Ref. level  0.63 | 0.55-0.73 | **< 0.001** |
| Yes | 1338 (57.9) | 938 (68.5) |  |  |  |
| **Diabetes mellitus, n (%)** | | | | | |
| No | 1693 (73.3) | 924 (67.5) | Ref. level  0.76 | 0.66-0.88 | **< 0.001** |
| Yes | 618 (26.7) | 445 (32.5) |  |  |  |
| **Previous thromboembolic event, n (%)** | | | | | |
| No | 1990 (86.1) | 1210 (88.4) | Ref. level  1.23 | 1.00-1.50 | **0.047** |
| Yes | 321 (13.9) | 159 (11.6) |  |  |  |
| **Vascular disease, n (%)** | | | | | |
| No | 1511(65.4) | 838 (61.2) | Ref. level  0.84 | 0.73-0.96 | **0.011** |
| Yes | 800 (34.6) | 531 (38.8) |  |  |  |
| **AF type: Non-permanent, n (%)** | | | | | |
| No | 720 (31.2) | 657 (48.0) | Ref. level  2.04 | 1.78-2.34 | **< 0.001** |
| Yes | 1591 (68.8) | 712 (52.0) |  |  |  |
| **CHA_2_DS_2_-VASC score**  **mean (SD)** | 3.9 (1.9) | 4.0 (1.8) | 0.98 | 0.94-1.02 | 0.254 |
| **HASBLED score**  **mean (SD)** | 2.0 (0.9) | 2.0 (1.0) | 0.89 | 0.83-0.96 | **0.002** |
| **eGFR** | 55.8 (16.1) | 53.3 (16.4) | 1.01 | 1.00-1.01 | **< 0.001** |
| eGFR ≥ 60 ml/min/1.73m^2^, n (%) | 869 (37.6) | 455 (32.9) | Ref. level  0.83 | 0.72-0.95 | **0.008** |
| eGFR < 60 ml/min/1.73m^2^, n (%) | 1442 (62.4) | 913 (67.1) |  |  |  |
| **LA diameter**  **mean (SD)** | 46.1 (7.3) | 48.8 (8.1) | 0.95 | 0.94-0.96 | **< 0.001** |
| **Antiplatelet drug/drugs, n (%)** | | | | | |
| No | 2169 (93.9) | 1236 (90.3) | Ref. level  0.61 | 0.48-0.78 | **< 0.001** |
| Yes | 142 (6.1) | 133 (9.7) |  |  |  |

Data are presented as number (percentage) or mean (standard deviation) (SD) or median (interquartile range) (IQR); Abbreviations: AF, atrial fibrillation; eGFR, estimated Glomerular Filtration Rate; LA, left atrium; NOAC, non-vitamin K antagonist oral anticoagulant; OAC, oral anticoagulation therapy; VKA, vitamin K antagonist oral anticoagulants
